# PROTOCOL: Interventions to Promote Inclusive Governance for Underserved Population in Sub‐Saharan Africa: An Evidence and Gap Map

**DOI:** 10.1002/cl2.70025

**Published:** 2025-03-07

**Authors:** Clarice Panyin Nyan, Sheila Agyemang Oppong, Takyiwaa Manuh, David Sarfo Ameyaw

**Affiliations:** ^1^ International Centre for Evaluation and Development Tema Accra Ghana

**Keywords:** Evidence and Gap Map, inclusive governance, policy intervention, sub‐Saharan Africa, underserved population

## Abstract

This Protocol for an Evidence and Gap Map (EGM) aims to identify, map, and provide an overview of the existing evidence and gaps in inclusive governance interventions for underserved populations in sub‐Saharan Africa. The specific objectives are as follows: (1) identify evidence clusters that present opportunities for evidence synthesis and (2) identify evidence gaps that require additional studies, research, and evaluations.

## 1 Background

### 1.1 Introduction

#### 1.1.1 The Problem, Condition, or Issue

The United Nations' (UN) Sustainable Development Goals (SDGs) place emphasis on the establishment of inclusive, effective, and accountable institutions at all levels through fostering public sector integrity, anti‐corruption efforts, and trust to prevent policy capture. While good governance is widely acknowledged as a crucial factor in global development, governance metrics depict various challenges. According to Freedom House, the number of free countries worldwide reached its lowest point in 18 years (Freedom House 2021). In Africa, the overall governance score was noted to have flatlined since 2019, and in 2021, much of Africa was less safe, secure, and democratic than in 2012, according to the latest Ibrahim Index of African Governance (Mo Ibrahim Foundation 2020). Furthermore, Transparency International (Barometer 2016) found that nearly 40% of the population in Asia and the Pacific perceived public services, such as law enforcement, to be highly corrupt. There is a pervasive phenomenon of elite capture in numerous countries whereby institutions responsible for managing public resources have been compromised. This capture is perpetuated by existing social, political, and economic power imbalances that predominantly benefit a privileged minority within society. Such imbalances systematically marginalize entire populations based on factors such as gender, place of birth, social class, ethnicity, or other aspects of their identity.

Increasingly, there is a shift in focus from good governance to inclusive governance. Inclusive governance entails providing marginalized populations with the opportunity to have their needs, voices, and interests acknowledged. Additionally, it empowers them to exercise their influence by organizing themselves and impacting political decision‐making. The objective is to prioritize decisions that serve the common good rather than specific sectarian interests. Numerous civil society organizations (CSOs), including those that represent impoverished individuals, play a vital role in mobilizing and supporting underprivileged populations to actualize their potential for change.

However, government agencies and even CSOs frequently encounter several challenges that hinder their ability to efficiently provide public goods and services or to track and monitor performance. Public contracting and procurement processes are often not transparent or inclusive, while the coordination and effectiveness of citizen participation in lower‐level planning procedures may be non‐existent or weak. These issues of coordination and effectiveness have a particularly adverse impact on marginalized communities, such as youth, women, people with disabilities, ethnic minorities, and the urban poor. While several countries claim to have established mechanisms to involve the public in policy development and decision‐making, these mechanisms often suffer from poor implementation. Underserved communities face a lack of access to government information, media coverage, and lobbying efforts on issues that directly impact them. Moreover, there is a dearth of data‐driven decision‐making and meaningful participation for marginalized communities in expanding services. Some governments and CSOs are actively exploring diverse initiatives to foster inclusive governance outcomes that effectively serve disadvantaged communities. These endeavors seek to address current limitations and deficiencies while upholding the commitments and targets set at regional and international levels. Thus, in previous studies, the emphasis has been placed on good governance. However, this evidence and gap map (EGM) will significantly contribute to the growing body of knowledge regarding inclusive governance interventions. By doing so, it will offer valuable insights into how these interventions can effectively amplify the voices of underserved populations and address their specific needs.

#### 1.1.2 Why It is Important to Develop the EGM

To make informed decisions that will have an impact on inclusive governance and identify gaps in the existing literature, it is crucial to conduct a thorough study of the topic (Snilstveit et al. 2016). Currently, some evidence is available on different interventions implemented by organizations to enhance inclusion for underserved populations. However, this valuable information is dispersed across various databases, making it challenging to make well‐informed decisions. An Evidence Gap Map (EGM) serves the purpose of consolidating all the relevant information and evidence in one centralized location, providing a visual summary that enables decision‐makers to obtain a comprehensive overview of the available evidence (Snilstveit et al. 2016). Moreover, the EGM also identifies gaps in the existing evidence, highlighting areas that require further research.

However, we have not identified any registered protocols or published EGMs pertaining to our proposed EGM. A thorough search of multiple databases conducted by two independent researchers has yielded no relevant results. Notably, two EGMs conducted by Berretta et al. (2021) and Thota et al. (2022) examined inclusion for civil society and inclusion for children living with disabilities, respectively, which were somewhat like our proposed EGM, but on a more limited scale. Therefore, our EGM aims to fill this knowledge gap.

### 1.2 Conceptual Framework

The conceptual framework of this EGM explains how the interventions mentioned below are hypothesized to affect the four outcomes of interest: movement and coalition activities and achievements; independent and pluralistic Media; use of key government information; and policy and policymakers' attitudes and behaviors. The framework demonstrates how inclusive governance for underserved populations leads to short or intermediate‐term outcomes, which eventually result in long‐term outcomes. This framework explains six inclusive governance interventions: the first is grassroots movements; this intervention includes developing and strengthening grassroots movements and coalitions, mobilizing resources for movements and coalitions, and collective actions and campaigns. The second is Civil Society Organizations advocacy: This intervention involves developing local philanthropic networks and initiatives, alliance building, advocacy and lobbying, and active participation of women and youth in coalitions and movements.

The third area is public policy and government agencies: interventions in this area include inclusive and responsive governance frameworks, creating awareness of public policies and governance mechanisms, and providing support for domestic work. The fourth area is media: interventions in this area include the liberalization of media spaces, diversified media programs, the elevation of voices of underserved populations, and the fostering of a gender‐sensitive culture in media. The fifth area is access to information: interventions in this area include making local and national information accessible to underserved populations, ensuring that official information and documents are available in local languages, and implementing a right‐to‐information policy.

**Figure 1:**
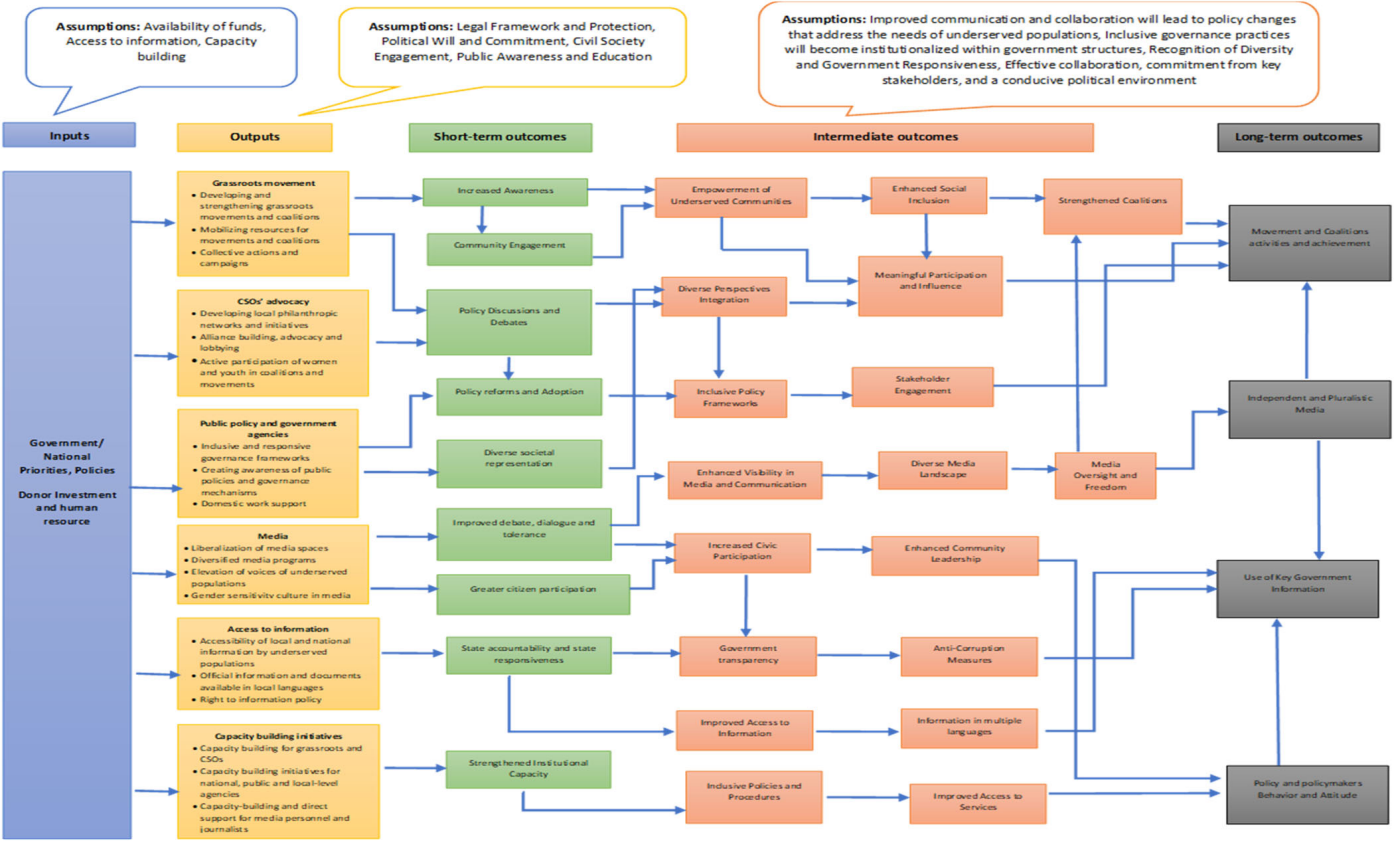
Conceptual framework.

The sixth area is capacity‐building initiatives: interventions in this area include providing capacity building for grassroots and Civil Society Organizations (CSOs), capacity‐building initiatives for national, public, and local‐level agencies, and offering capacity‐building and direct support for media personnel and journalists (Figure 1).

Interventions aimed at grassroots movements include developing and strengthening grassroots movements and coalitions, mobilizing resources, and undertaking collective actions and campaigns. These interventions aim to achieve short‐term outcomes, including increased awareness among underserved populations regarding the importance of inclusivity in governance and its impact on diverse communities. This heightened awareness can contribute to a more inclusive mindset and approach to decision‐making, leading to increased community engagement. Underserved populations become more involved in community events, forums, and activities, indicating a growing sense of community empowerment and involvement. Furthermore, grassroots movements can stimulate policy discussions and debates among policymakers, experts, and the public. These discussions may take the form of forums, roundtable discussions, or hearings focused on advocacy issues. As a result of increased awareness and community engagement, underserved communities can experience empowerment (Chetkovich and Kunreuther 2006). The strengthened coalitions resulting from enhanced social inclusion and meaningful participation and influence by these underserved populations contribute to long‐term outcomes in movement and coalition activities and achievements. Moreover, grassroots movements can promote independent and pluralistic media and facilitate access to key government information.

Advocacy interventions by CSOs include various strategies such as developing local philanthropic networks and initiatives, building alliances, engaging in advocacy and lobbying, and encouraging the active participation of women and youth in coalitions and movements. The immediate outcome of this intervention is the facilitation of policy discussions and debates, where CSOs can make their voices heard by establishing networks, platforms, and collaborations with large groups. Simultaneously, decision‐makers are more likely to take action when they see that the problem is supported by the broader public, leading to policy reforms and adoption (Katito and Aggad 2009). As a result of CSOs' advocacy efforts, policy discussions and debates will promote the integration of diverse perspectives and the meaningful participation and influence of underserved populations in decision‐making processes. This, in turn, contributes to the long‐term success of movements and coalition activities (Ghaus‐Pasha 2005).

Public policy and government agencies utilize various interventions aimed at inclusive and responsive governance frameworks, raising awareness of public policies and governance mechanisms, and providing support for domestic work. These interventions have the potential to bring about policy reforms, promote diverse societal representation, and increase the participation of marginalized populations in decision‐making processes at all levels of government. By considering the needs and voices of the people when designing, delivering, implementing, and evaluating public policies and services, governments can directly involve citizens in decision‐making and utilize data analysis to assess policy performance and anticipate future needs (Charbit 2011). Ultimately, these efforts contribute to the development of inclusive policy frameworks and stakeholder engagement, building support for government policies. In the long term, these interventions lead to the mobilization of movements and coalitions, as well as the utilization of key government information to achieve desired outcomes.

Media interventions include the liberalization of media spaces, diversified media programs, the elevation of voices of underserved populations, and the promotion of gender‐sensitive culture in media. When these media interventions are in place, they lead to improved debate, dialog, tolerance, and greater citizen participation in the media space as short‐term outcomes. This, in turn, enhances visibility in media and communication and promotes government transparency. It also creates a diverse media landscape and enhances community leadership for underserved populations. Additionally, the media plays a crucial role in shaping society. They connect citizens with developments around them and with the prevailing social, economic, cultural, and political institutions. The media also provide channels for these institutions to interact with citizens. Through media oversight and freedom, there will be free, independent, and pluralistic media based on freedom of information and expression, which are core elements of any functioning democracy. Freedom of the media is, in fact, essential for the protection of all other human rights. Instances of torture, discrimination, corruption, or misuse of power often become known because of the work of investigative journalists. Making the facts known to the public is often the first essential step in addressing human rights violations and holding governments accountable. In the long term, this leads to the establishment of independent and pluralistic media and the utilization of key government information.

Access to information, including local and national information, official documents, and information in local languages, is crucial for underserved populations. These interventions aim to promote state accountability, state responsiveness, government transparency, and improved access to information for underserved populations (Mansuri and Rao 2012). Ultimately, this can result in anti‐corruption measures and the availability of state information in multiple languages, ensuring that underserved populations can understand government processes. Over the long term, when underserved populations have access to government information, it can lead to the utilization of key government information and influence the behavior and attitudes of policymakers (McGee and Gaventa 2011).

Capacity‐building initiatives comprise capacity building for grassroots and CSOs, as well as capacity‐building initiatives for national, public, and local‐level agencies. Additionally, there is capacity‐building and direct support for media personnel and journalists. Implementing these capacity‐building initiatives leads to strengthened institutional capacity for CSOs, agencies, and grassroots movements. Consequently, this results in inclusive policies and procedures and improved access to services. Over time, these efforts can also lead to a change in the behavior and attitude of policymakers.

## 2 Objectives

The aim of this EGM is to identify, map, and provide an overview of the existing evidence and gaps in inclusive governance interventions for underserved populations in sub‐Saharan Africa. The EGM will specifically:

- Identify which evidence clusters present opportunities for evidence synthesis.
- Identify the evidence gaps that require additional studies, research, and evaluations.

## 3 Methods

Our EGM will adhere to the methodology outlined by the Campbell Collaboration for developing evidence and gap maps, as stated by White et al. (2020).

### 3.1 Defining an EGM

There are multiple definitions of an Evidence‐Based Map (EGM) in the literature, including those by White et al. (2020) and Snilstveit et al. (2013). However, based on White et al. (2020), we define an EGM as a “systematic evidence synthesis product which displays the available evidence relevant to a specific research question.” The evidence is obtained through a search using a predetermined, published search protocol. In addition to the map, the study will include a descriptive report that summarizes the evidence and identifies gaps. This report will be used by various stakeholders, including researchers, research commissioners, policymakers, and practitioners (Saran and White 2018). Unlike systematic reviews, we will not synthesize the results. The proposed EGM will serve as a platform for a body of evidence on interventions for inclusive governance outcomes.

### 3.2 Scope of EGM

The scope of this proposed Evidence and Gap Map (EGM) will be guided by the Population, Intervention, Comparison, Outcomes, and Study Designs (PICOS) framework. It will encompass studies that demonstrate a relationship between interventions for underserved populations (see Table 1) and inclusive governance outcomes (see Table 2). The EGM will evaluate and include studies that examine the effects of interventions on the outcomes of interest using quantitative (i.e., experimental, quasi‐experimental, and nonexperimental) and systematic review methods. In addition to conventional EGM approaches, we will also incorporate studies employing qualitative research designs. This EGM will cover studies published from 1990 onwards for two primary reasons. Firstly, a preliminary search indicates that many relevant studies emerged after 1990. Conducting an unrestricted search might result in an excess of studies that are not directly relevant, thereby increasing the screening workload for the team. Secondly, our focus is specifically on assessing the evidence and identifying gaps within recent literature.

**Table 1:** Intervention categories and sub‐categories.

Intervention category	Sub‐categories	Description and examples
Grassroots movements		Highlights the development and support to grassroots social movements, including their organization, campaigns, and resource mobilization.
	Developing and strengthening grassroots movements and coalitions	Interventions aimed at structuring and supporting the development of grassroots movements and coalitions to advance their objectives and address social, political, or environmental issues.
	Mobilizing resources for movements and coalitions	Securing the necessary assets, both financial and non‐financial, to support the activities and objectives of social movements and coalitions. This typically involves strategies and actions aimed at obtaining funding, expertise, and support from various sources, including individuals, organizations, and government agencies.
	Collective actions and campaigns	A grassroots campaign is one that uses the people in a given district, region, or community as the basis for a political or economic movement. Grassroots movements and organizations use collective action from the local level to implement change at the local, regional, national, or international levels. Forming alliances or partnerships between diverse individuals, groups, or organizations to undertake strategic efforts or advocate to bring about awareness, change, or action of government responsiveness to their needs.
CSOs' advocacy		This category focuses on advocacy efforts and initiatives of CSOs aimed at promoting inclusive governance. This includes activities related to philanthropic networks, alliance building, and ensuring the participation and influence of youth and women through advocacy and lobbying.
	Developing local philanthropic networks and initiatives	Efforts to establish and strengthen networks of philanthropic organizations and individuals at the community or regional level. These initiatives aim to foster collaboration, resource sharing, and strategic giving to address local challenges and support community development.
	Alliance building, advocacy, and lobbying	A collaborative partnership or network formed among multiple Civil Society Organizations (CSOs) enabling them to combine their efforts, share resources, and amplify their collective impact, ultimately enhancing their ability to address and influence significant social, political, or economic issues. Developing advocacy campaigns and lobbying efforts to influence policy changes that benefit underserved populations.
	Active participation of women and youth in coalitions and movements	Active engagement and the impact women and youth have within the collective efforts to bring about social, political, or cultural change. E.g. Leadership roles, advocacy efforts, and decision‐making within the coalitions and movements.
Public policy and government agencies	Inclusive and responsive governance frameworks	Having a governance framework and approach that prioritizes the active involvement of diverse stakeholders, particularly marginalized or underrepresented groups, in the decision‐making and policy development processes of a government or institution.
	Creating awareness of public policies and governance mechanisms	Creating awareness to influence the laws, policies, and procedures that regulate how governments run, how resources are distributed, and how public services are provided. For stakeholders, including grassroots movements, women, and youth, to actively participate in the governance of their societies and hold governments accountable, they must be aware of public policies and governance procedures.
Media	Liberalization of media spaces	Interventions designed to make media spaces accessible to a growing number of users and also to increase broadcasting at all levels. **E.g.** setting up of local radio stations.
	Diversified media programs	Strategies that seek to offer a variety of content and programs in many formats and platforms to increase the coverage and range of issues.
	Elevation of voices of underserved populations	Ensuring the coverage of content of particular relevance and interest to underserved populations and increasing their access to and control of media spaces to bridge knowledge and participation gaps.
	Gender sensitivity culture in media	Interventions to support the development of a culture of gender/GESI sensitivity and safeguarding within the media space, with a special focus on women, young persons, and PWDs.
Access to information	Accessibility of local and national information by underserved populations	Actions to increase access to and understanding of official information and data to enable underserved populations to participate actively in national and civic life. (Are made possible by access to information for people, lawmakers, researchers, and the media. It is crucial for encouraging openness, responsibility, and knowledgeable judgment in a democratic society).
	Official information and documents available in local languages	This approach aids in ensuring that all individuals, including those who might not be fluent in the official or dominant language, have access to information on policies, programs, and services provided by the government in local languages.
	Right to information policy	A system of rules, laws, or regulations that control the public's right to access data and records maintained by the government. By enabling people (underserved populations), journalists, researchers, and organizations to seek and access information stored by government agencies and public bodies, with the aim of advancing transparency, accountability, and openness in government.
Capacity‐building initiatives	Capacity building for grassroots and CSOs	Efforts aimed at enhancing the skills, knowledge, resources, and organizational capacity of marginalized groups, CSOs, grassroots organizations, and collective movements and alliances. Examples: training programs, leadership development, strategic planning.
	Capacity‐building initiatives for national, public, and local‐level agencies	Activities and initiatives designed to increase the resources, capabilities, and expertise of government agencies and organizations. These initiatives are essential for enhancing the efficiency and responsiveness of state institutions and agencies in providing public services, carrying out policies, and attending to the requirements of their citizens.
	Capacity‐building and direct support for media personnel and journalists	Interventions aimed at helping county‐based journalists adopt evidence‐based data journalism on issues affecting underserved populations in their diversity.

**Table 2:** Outcome categories and sub‐categories.

Outcome category	Sub‐categories	Description and examples
Movements and Coalitions, and CSO's activities and achievements		Strategies aimed at enhancing their effectiveness, resilience, and ability to achieve their goals. The process of empowering these collective entities to better advocate for their causes, mobilize support, and drive positive change.
	Funding to movements and coalitions	Securing adequate financial resources and support for social or advocacy movements and coalitions. This outcome signifies the success of efforts to raise funds and resources to sustain the activities and advocacy work of these collective efforts.
	Participation of local communities in the activities and campaigns of movements and coalitions	Active engagement and involvement of community members, particularly women and youth, in the various initiatives, actions, and advocacy efforts led by social or advocacy movements and coalitions.
	Influence of Grassroots and CSOs on public policies	Changes in policy from or as a result of advocacy.
	Civic awareness	Awareness and knowledge of civic rights and responsibilities.
	Partnerships	Measures of collaboration and resource sharing. Also measures of diverse networking for civic engagement.
Civic participation	Civic participation in coalitions and movements	Participation of underserved populations in movements and coalitions
	Civic participation in governance	Representation and participation of underserved groups in government positions, policy, and decision‐making process
	Social, cultural, and political change	Measures of women and youth in leadership roles (grassroots movements, CSOs, media, and policymakers)
Capacity	Capacity of grassroots movements, CSOs, media, and policymakers	Measures of skills, knowledge, awareness, and organizational capacity
Independent and Pluralistic Media		A diversity of media providers, a variety of opinion‐forming media content within a medium, or a combination of both to enable a free and democratic public opinion‐forming process, regardless of the form of a medium's offer. It is intended to both monitor government and reflect the perspectives of underserved populations.
	Enhanced Civic Space	Civic space is the respect for the freedoms of association, peaceful assembly, and expression in policy, legislation, and practice, as well as the degree to which the state upholds fundamental rights. It further encompasses the freedom to access, gather, and share knowledge and ideas of all types across boundaries and through all mediums.
	Media coverage of underserved groups	Media coverage is the attention and exposure that a person, brand, event, or topic receives across a variety of media outlets, including print, broadcast, and online. It covers all types of information that highlights and discusses the topic, including news pieces, interviews, features, reviews, and other types of content that are relevant to underserved groups.
Use of Key Government Information		Outcomes that enable underserved populations to use government data and information to support their activities and aspirations. The use of key government information that includes data, documents, reports, statistics, and other forms of information generated or collected by government agencies and bodies at various levels of government by underserved populations.
	Global Right to Information Rating	The Global Right to Information is the leading global tool for assessing the strength of national legal frameworks for accessing information held by public authorities (i.e., the right to information or RTI). It is widely used by inter‐governmental organizations, RTI advocates, government, legislators, lawyers, academics, and others. https://www.rti-rating.org
	Rule of Law Index	The Rule of Law Index is a quantitative assessment tool designed to offer a comprehensive picture of the extent to which countries adhere to the rule of law. https://www.oecd.org/mena/governance/45447873.pdf
	Public participation in budgets	Participatory budgeting is a form of citizen participation in which citizens are involved in the process of deciding how public money is spent. Local people are often given a role in the scrutiny and monitoring of the process following the allocation of budgets.
	Corruption Perception Index	The Corruption Perceptions Index (CPI) is an index which ranks countries “by their perceived levels of public sector corruption, as determined by expert assessments and opinion surveys.” The CPI generally defines corruption as an “abuse of entrusted power for private gain.” https://www.transparency.org/en/cpi/2021
	Global Open Data Index	The Global Open Data Index tracks whether data is actually released in a way that is accessible to citizens, media and civil society and is unique in crowd‐sourcing its survey of open data releases around the world.
Policy and policymakers	Knowledge, attitude, or behavior of policymakers, the public, or other key actors	Measurable and demonstrable changes in the understanding, perceptions, or actions of policymakers, the public, or other key actors who play a crucial role in public policy. Examples: Knowledge Outcome: demonstrable increases in awareness, understanding, or knowledge of the needs of underserved populations among policymakers, the public, or other relevant stakeholders. Attitude Outcome**—**Measurable shifts in the attitudes, beliefs, or perceptions of key actors on issues regarding the underserved. Behavior Outcome**—**Observable changes in the actions, decisions, or behaviors of policymakers, the public, or other influential actors toward their responsiveness to the needs of underserved populations.

#### 3.2.1 Population

This EGM focuses on underserved (marginalized) groups in sub‐Saharan African (SSA) countries and the institutions that provide services for them. The specific subgroups within the marginalized population include women (including those from rural areas, ethnic minorities, and informal settlements in urban areas, such as market women), peasant/smallholder farmers, youth (including young boys and girls from rural areas, urban informal settlements, and young girls from ethnic minority communities), persons living with disabilities (PWDs), ethnic and sexual minorities, and the Fulbe/fulani (the Fulbe are an ethnic group of people spread across many countries, predominantly in West Africa and are considered the largest ethnic group in the sub‐region) community. The organizations that provide services to these marginalized groups, which will be considered for this EGM, include civil society organizations (CSOs), policymakers, grassroots movements, and media actors.

#### 3.2.2 Intervention

The map will cover interventions carried out by grassroots movements, CSOs, media, and policymakers. Its objective is to empower the underserved population to advocate for government policies that are tailored to their needs and promote their participation in governance. Additionally, the map will include interventions aimed at assisting grassroots movements, CSOs, policymakers, and media in delivering services to the underserved. Table 1 offers a clear summary of the interventions included in this EGM.

#### 3.2.3 Comparison

There is no specified comparison group in this EGM. However, the EGM will feature studies such as impact studies and comparison studies, which analyze different cases to identify similarities or differences.

#### 3.2.4 Outcomes

This EGM will prioritize five key outcomes, which include the following: the provision of resources to grassroots initiatives, civil society organizations (CSOs), media outlets, and policymakers; the enhancement of capacity among underserved populations, grassroots movements, CSOs, media outlets, and policymakers; the promotion of an independent, liberalized, and pluralistic media landscape; the facilitation of access to government information for the underserved; the active involvement of the underserved in policy‐making processes; and finally, the dissemination of knowledge and awareness among the underserved population, grassroots movements, CSOs, media outlets, and policymakers. For a concise description of these outcomes, please refer to Table 2.

#### 3.2.5 Study Design

This EGM will feature a comprehensive compilation of published and unpublished reports, studies, and reviews. It will encompass both experimental and nonexperimental studies, covering a wide range of research areas. Included in this EGM will be various types of studies, such as effectiveness studies, nonexperimental quantitative studies, process evaluations, summative evaluations, qualitative studies, systematic and scoping reviews, and meta‐analysis of eligible studies.

#### 3.2.6 How We Plan to Handle Adverse Outcomes

This EGM will examine studies in which interventions for inclusive governance resulted in adverse outcomes. This approach is implemented to ensure a balanced reporting of intervention effects, mitigating the tendency to report only positive outcomes (White et al. 2020). The EGM will categorize adverse outcomes, like positive outcomes, according to the specific outcome they pertain to, ensuring neutrality in the outcomes assessed.

### 3.3 Other Inclusion and Exclusion Criteria for Eligible Studies

Our criteria for including and excluding studies critically follows our PICOS (Population, Intervention, Outcomes, and Study design) approach, built upon the intervention‐outcome strategy (Saran and White 2018). We will assess the eligibility of the included studies (i.e., published, and unpublished) (White et al. 2020).

#### 3.3.1 Inclusion Criteria

##### 3.3.1.1 Type of Studies

Ongoing and completed experimental, nonexperimental, qualitative studies, studies with modeling designs, systematic and scoping reviews, and eligible evaluation studies (impact evaluation, process evaluation, formative evaluation, and summative evaluation) will be included. Peer‐reviewed articles, preprint peer‐reviewed articles, reports, conference papers, working papers, discussion papers, dissertations, and protocols will also be added to our EGM.

##### 3.3.1.2 Geographical Location

Studies focused on sub‐Saharan African countries that target underserved populations, including rural women, ethnic minority communities, women in informal urban settlements, youth in rural areas and urban informal settlements, young girls from ethnic minority communities, persons living with disabilities (PWDs), sexual minorities, the Fulbe community, civil society organizations (CSOs), journalists, and politicians.

##### 3.3.1.3 Timeframe

The eligible studies for inclusion in this EGM will encompass the period between 1990 and 2023, regardless of their publication status. This timeframe has been selected due to the heightened emphasis on fostering inclusive governance during the 1990s, exemplified by significant developments such as the dismantling of apartheid in South Africa (Mapadimeng 2013). By adopting this specific timeframe, we aim to comprehensively capture all pertinent research pertaining to inclusive governance in sub‐Saharan Africa within our search results.

##### 3.3.1.4 Language

Our EGM will consider all studies written in English and French. In addition, French‐written studies will be included as our research team comprises a French speaker who will apply the screening protocol and code the selected studies.

#### 3.3.2 Exclusion Criteria

Studies conducted in languages other than English and French, as well as studies conducted outside of the sub‐Saharan region, will be excluded from consideration.

Additionally, this EGM will not include studies conducted before the year 1990. Studies that do not focus on interventions to promote inclusive governance for underserved populations in sub‐Saharan Africa, specifically on movement and coalition activities and achievements, the utilization of key government information, policies, and policymakers, and the independent and pluralistic media, will also be excluded from this EGM.

### 3.4 Search Strategy, Search Terms, and Screening

#### 3.4.1 Search Strategy

With the assistance of an information specialist, we will implement a comprehensive search strategy designed to identify all potential studies, both published and unpublished, for inclusion in this Evidence and Gap Map (EGM). This approach aims to minimize publication bias. The search will be conducted across four pertinent scientific and academic databases: CAB Abstract, Scopus, Web of Science (Web of Science Core Collection Citation Database), and Dimensions. Additionally, we will explore additional potential papers using OpenAlex within the Eppi‐reviewer software (Thomas and Brunton 2010). OpenAlex functions as an index containing vast interconnected entities within the global research system (Priem et al. 2022). We will further expand our search scope to search for studies and papers/reports from gray literature, including websites and other organizational databases/websites such as the following:

- EPPI knowledge library
- Social Science Research Network (SSRN)
- Centre for Strategic and International Studies
- Abdul Latif Jameel Poverty Action Lab
- Innovations for Poverty Action (IPA)
- Evidence in Governance and Politics (EGAP)
- National Bureau of Economic Research (NBER)
- USAID‐ Development Experience Clearinghouse (DEC)
- World Bank Economic review
- World Bank documents and reports
- The Cochrane Central Register of Controlled Trials (CENTRAL)
- The Campbell library
- Cochrane library
- International Initiative for Impact Evaluation (3ie)
- World Bank ‐ Independent Evaluation Group (IEG)
- African Development Bank ‐ Evaluation reports
- Asian Development Bank ‐ Evaluation resources
- Google scholar
- Freedom House
- Organization for Economic Co‐operation and Development
- Institute of Development Studies (IDS) InfoDEV

We will further conduct hand searches in these journals:

- Journal of Management and Governance
- Journal of Good Governance and Sustainable Development in Africa
- Governance
- Pan African Journal of Governance and Development
- International Journal of Electronic Governance
- International Journal of Disclosure and Governance
- Sabinet African Journals
- Policy commons

To ensure the comprehensive inclusion of all relevant evidence in the EGM, we will employ a rigorous backward‐track citations approach. This approach will enable us to identify primary studies, reviews, and meta‐analyses that may not have initially been captured in our search but are still eligible for inclusion. Our focus will be directed toward authors who have been extensively cited within the relevant subject field. Additionally, we will conduct thorough searches of registries and repositories to identify ongoing and completed studies, reviews, and EGMs. The inclusion of ongoing studies will provide valuable insights into emerging research, thereby informing future updates to the EGM. To maintain the utmost relevance, we will implement a decision rule that confines our search to a timeframe of no more than 5 years from the registration date. This will prevent the inclusion of any ongoing studies that are significantly overdue.

Additionally, there will be access to further research through evaluation reports, university theses, and other forms of gray literature. We will also incorporate potential research from relevant EGMs that have been conducted by organizations such as 3ie and Campbell Collaboration into our EGM.

Every eligible manuscript will be converted into a Research Information System (RIS) and uploaded into the EPPI‐Reviewer application (Thomas and Brunton 2010). To meet reporting requirements, we will keep a record of our literature search operations.

#### 3.4.2 Search Terms

We intend to incorporate studies with various study designs, including qualitative studies and process evaluations. As a result, the search terms for this evidence generation method (EGM) will not be limited to any specific study design; instead, study designs will be utilized as filters when presenting the EGMs. For instance in running the search in SCOPUS, a decision will be made based on the number of search results from running the search using the study design or not. We will create search terms based on the PICOS framework to retrieve published studies or reports from electronic databases (see Supporting Information S1: Appendix 1 for search terms examples with an illustration of search results in SCOPUS).

#### 3.4.3 Screening

**Figure 2:**
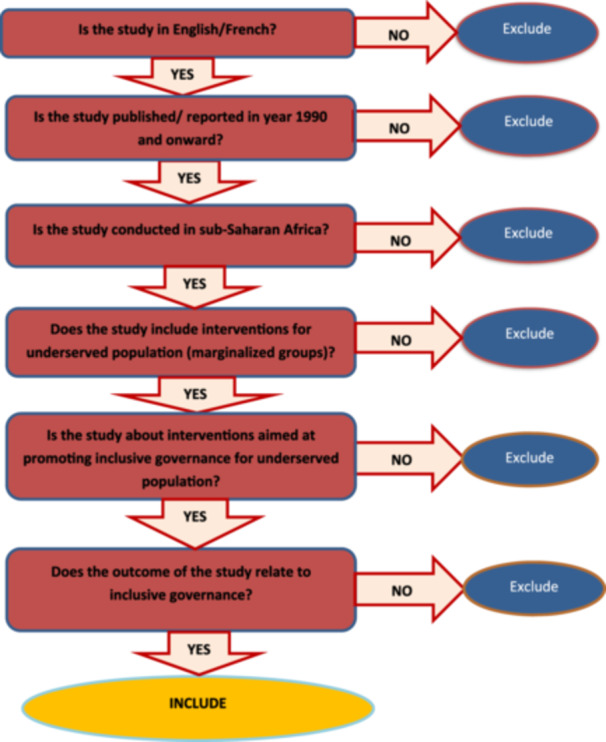
Screening tool to assess eligible studies.

The EPPI‐Reviewer software will be utilized to identify and eliminate any duplicate studies or papers before commencing the screening process. To optimize the screening process, a machine learning model will be implemented to prioritize the identified papers based on their relevance and importance in accordance with the eligibility criteria for the proposed Evidence and Gap Map (EGM) review. The eligibility screening for both published and unpublished studies or papers for the EGM will be carried out in two stages. Firstly, the title and abstract of the studies will be screened using predetermined eligibility criteria (screening tool), as shown in Figure 2. Subsequently, a full‐text screening will be conducted for the studies that have successfully passed the title and abstract screening stage. All studies will be independently reviewed by two reviewers. In the event of any disagreements regarding the inclusion of a paper in the EGM, the reviewers will collaborate, and, if necessary, a third reviewer will conduct an independent screening to resolve any discrepancies.

### 3.5 Data Extraction (Coding), Cleaning, and Analysis

#### 3.5.1 Coding

An extraction form will be utilized for the purpose of data extraction. This form will present pertinent details such as region, country, language, population, study status, intervention, outcome, study design, and evaluation type. To facilitate data management, analysis, and coding, EPPI‐Reviewer (Thomas and Brunton 2010) will be employed. To ensure accuracy, duplicate data will be gathered for comparison purposes. Each study or paper will then be coded in EPPI‐Reviewer based on a pre‐established form (See Appendix 2 for the Coding form).

#### 3.5.2 Data Cleaning

To ensure the accuracy of the information provided in the EGM, the data coded in the EPPI reviewer will be cleaned before analysis. Researchers will conduct univariate analysis to identify any outliers in the codes. If any outliers are found, they will be investigated and examined to determine the cause. Any errors that are identified will be corrected before proceeding with the analysis. Additionally, any missing studies will be searched for, and if necessary, the authors will be contacted.

#### 3.5.3 Data Analysis Plan

Various entries will be recorded in the EGM to encompass the studies encompassed by the EGM (White et al. 2020). Studies that comprise systematic reviews with multiple interventions and outcomes will be regarded as a single study. However, each intervention will be assigned a separate code in the EGM. For studies that feature interventions both within and outside the EGM's scope, only the pertinent studies will be incorporated, while interventions outside the scope will be excluded. In the case of studies with different published versions, such as manuscripts or working papers, the most recent publication will be taken into account (Malhotra et al. 2021).

The EPPI‐Reviewer software will be utilized to analyze the studies included in the EGM. EPPI‐Reviewer is a screening tool developed by reputable systematic review groups (Tsou et al. 2020). The analysis will involve the use of summary statistics such as frequencies and percentages. The findings will be presented in graphs, tables, and cross tabulations, demonstrating the relationships between interventions, outcomes, regions, and study design. A comprehensive summary and synthesis of the evidence will be conducted to identify patterns and identify any gaps. Furthermore, the EGM's utility and limitations will be discussed.

For the creation of the EGM, both the EPPI‐Reviewer and Mapper software will be employed. The data will be coded and analyzed using these tools, and the development of the EGM will be informed. The frequencies established in the evidence will be visually represented in the EGM using bubbles of varying sizes. The size of each bubble will correspond to the frequency of a particular outcome or intervention.

#### 3.5.4 Presentation of EGM

EGMs are represented graphically on a matrix, which consists of two main dimensions. The matrix primarily displays interventions and outcomes. In our EGM matrix, the eligible study designs are depicted using colored circles, as described by Lane et al. (2023). We will establish the map framework through collaborative discussions with key stakeholders such as the Hewlett Foundation and the Campbell Collaboration. The cells within the matrix will include filters to organize results based on variables such as interventions, outcomes, study designs, study status, country, region, and location.

### 3.6 Critical Quality Appraisal of Studies

To assess the methodological quality of systematic reviews, we will utilize the AMSTAR 2 tool (Shea et al. 2017). As we have limited time for the EGM, we will not evaluate the quality of the primary research. However, we will categorize and report the study designs used by the qualifying studies as filters for presenting the EGM (White et al. 2020).

### 3.7 Pilot Study of EGM

We will conduct a pilot study of our EGM with the goal of reducing the workload for team members and evaluating all components of the system, including our search strategy, screening process, and coding guidelines. To test the effectiveness of our coding form, we will utilize 20% of the eligible studies in the EPPI reviewer software. The row and column headings for interventions and outcome categories will be revised accordingly. Following this, a practicality test of the EGM will be carried out. Lastly, we will share the piloted EGM with stakeholders and gather their feedback on the system's features.

### 3.8 Stakeholder Engagement

To determine the scope and structure of the map, we conducted discussions with various stakeholders. This included the Gender Equity and Governance (GEG) team of the Hewlett Foundation, as well as their grantees and partners. We also engaged with AfroBarometer, the Center for Democratic Development (CDD) Ghana, experts from the African Population Health Research Council, researchers from the University of Ghana, the STAR Ghana Foundation, Women in Informal Employment: Globalizing and Organizing (WIEGO), and representatives from the Ministry of Finance and the Ministry of Gender, Children, and Social Protection. These meetings involved academics, researchers, and decision‐makers.

Throughout the entire process of conceptualizing, designing, and producing the EGM, we will closely collaborate with the Campbell Collaboration. The EGM will be a public good and will be published in an open‐access journal, ensuring accessibility for everyone. Our goal is for the findings of our EGM to have an impact on future decision‐making regarding research and funding priorities related to inclusive governance interventions for underserved populations in SSA. Specifically, we will examine civic participation, the use of key government information, and other outcomes of inclusive governance.

As the evidence and gap map is developed, these individuals will be consulted for feedback on the updated framework, preliminary findings, and draft map. Additionally, to gather input from a diverse range of stakeholders interested in inclusive governance, we will share the draft map and preliminary findings with them.

### 3.9 Plans to Update the EGM

The EGM will be periodically updated, typically every 2 years, based on the availability of funds and the findings of further research.

### 3.10 Sources of Technical and Financial Support

The Campbell Collaboration will collaborate with us to develop this EGM. They have a successful track record in producing EGMs and increasing EGM capacity. The Hewlett Foundation will offer financial assistance and technical advice from the funder's perspective to support the production of this EGM, while the Campbell Collaboration will provide technological support.

## Author Contributions

- **Content expertise**

David Ameyaw, the CEO of the International Center for Evaluation and Development (ICED), is the PI for this EGM. He has extensive experience in impact evaluation, project design and management, and EGMs, along with a strong research background.

Takyiwaa Manuh, an Emerita Professor at the University of Ghana, is the gender specialist for this EGM. She brings with her a wealth of experience in gender‐related issues.

Clarice Panyin Nyan, a PhD candidate at the University of Ghana, specializes in project management, evidence synthesis, and monitoring and evaluation.

Sheila Agyemang Oppong, a development economist specialist at ICED, contributes her expertise in economic and international development, EGMs, and issues related to women's economic empowerment and gender equality.

- **EGM methods expertise**
  David Ameyaw, Takyiwaa Manuh, Sheila Agyemang Oppong, and Clarice Panyin Nyan have experience in developing EGMs, building upon their previous collaborative work.
- **Information retrieval expertise**
  Rodney Malesi, a senior librarian from the United States International University, has been tasked with the responsibility of retrieving pertinent studies for the EGM. Rodney possesses exceptional expertise in literature retrieval and has successfully contributed to numerous systematic reviews and EGMs in the past. The authors will provide Rodney with the necessary assistance in accomplishing this objective.
- **Coding and data extraction**
  Sheila Agyemang Oppong and Clarice Panyin Nyan will be responsible for leading all coding and data extraction activities.
- **Statistical analysis**
  All team members possess the necessary educational and professional qualifications to perform statistical analysis and produce meaningful reports.

## Conflicts of Interest

The authors declare no conflicts of interest.

## Sources of Support

### Internal Sources

None.

### External Sources

This EGM is supported by the Hewlett Foundation.

## Supporting information

Supporting information.

## Data Availability

The authors have nothing to report.
