# Supplementary material for: PROTOCOL: Interventions to Promote Inclusive Governance for Underserved Population in Sub‐Saharan Africa: An Evidence and Gap Map
Source: Campbell Syst Rev. 2025 Mar 7;21(1):e70025. doi: 10.1002/cl2.70025 (PMC11888123; doi:10.1002/cl2.70025)
Supplement: Supplementary file 1 — Supporting information. [file CL2-21-e70025-s001.docx]

**Appendices**

**Appendix 1: Search terms**

| **Category (All Fields)** |  | **Search Terms** |
| --- | --- | --- |
| 1. **Population** | 1  SSA | “Angola” OR “Burundi” OR “Central African Republic” OR “Chad” OR “Democratic Republic of Congo” OR “Republic of Congo” OR “Rwanda” OR “Comoros” OR “Eritrea” OR “Ethiopia” OR “Kenya” OR “Madagascar” OR “Mauritius” OR “Seychelles” OR “Somalia” OR “South Sudan” OR “Sudan” OR “Tanzania” OR “Uganda” OR “Botswana” OR “Eswatini” OR “Formerly Known as Swaziland” OR “Lesotho” OR “Malawi” OR “Mozambique” OR “Namibia” OR “South Africa” OR “Zambia” OR “Zimbabwe” OR “Benin” OR “Burkina Faso” OR “Cabo Verde” OR “Cape verde” OR “Cameroon” OR “Cote d'Ivoire” OR “Ivory Coast” OR “Equatorial Guinea” OR “Gabon” OR “Gambia” OR “Ghana” OR “Gold Coast” OR “Guinea” OR “Guinea Bissau” OR “Liberia” OR “Mali” OR “Mauritania” OR “Niger” OR “Nigeria” OR “Sao Tome and Principe” OR “Senegal” OR “Sierra leone” OR “Togo” OR “africa south of the sahara” OR “sub-saharan Africa” OR “subsaharan Africa” OR “africa central” OR “central Africa” OR “africa southern” OR “southern Africa” OR “africa eastern” OR “east Africa” OR “eastern Africa” OR “africa western” OR “west Africa” OR “western Africa” OR “Africa” |
|  | 2  Underserved population | ((“Rural” OR “village” OR “margin*”) AND (“wom?n” OR "young” OR “youth”)) OR “Peasant farmers” OR “Small scale farmers” OR “Small holders” OR “Slum dwellers” OR “PWDs” OR “disabilit*” OR “Marginali?ed ethnic group” OR “ethnic minority” OR “sexual minority” OR “Lesbian” OR “Gay” OR “bisexual” OR “asexual” OR “transgender” OR “Two-Spirit” OR “queer” OR “intersex” OR “Fulani” OR “Fulbe” OR “fellata” OR “fula*” |
|  | 3  CSOs | “Non-governmental organization” OR “Civil society organization” OR “Non-profit Organizations” OR “Not-for-Profit Organizations” |
|  | 4  Media | ((“Journalists” OR “bloggers”) AND (“media” OR “press” OR “news”) AND (( “radio” OR “television” OR “newspaper”)) |
|  | 5  Politicians and civil service | “Politicians” OR “Policy makers” OR “decision makers” OR “legislators” OR “policy advisors” OR “state actors” OR “public officers” OR “civil servant” |
| **Population**  (All terms and fields) | **6** | #1 AND (#2 OR #3 or #4 OR #5) |
| **2 Interventions** | **7**  **Grassroots movement** | “movement” OR “ICT” OR “digitalization” OR “Digitalisation” OR “Capacity building” OR “Coalitions” OR “Community driven” OR “community based” OR “community” OR “Inclusive” OR “Policies” “grassroots” “campaigns" OR “organ*” OR “collective action” OR “ activism” |
|  | 8  CSOs advocacy | ((“CSO” OR “NGO” OR “non?profit organi?ation^ OR “Grassroot” OR “community”) AND (“advocacy” OR “alliance” OR “collective action” OR “lobbying” OR campaign”)) |
|  | 9  Public policy and government agencies | “Inclusive governance” OR “responsive governance” OR ((“awareness”) AND (“polic”* OR “governance”) |
|  | 10  Media | “Gender Sensitive” OR “media spaces” OR “diversified media” OR “Gender sensitivity” OR “local radio station” |
|  | **11**  **Access to information** | "information access" OR “access to information” OR “(“Language”AND “translation” AND “local”) OR "right to information" OR "liberali?ed media" OR "freedom of information" OR "open data" |
|  | **12**  **Capacity building initiatives** | ((“CSO” OR “Media” OR “Policy makers” OR “grassroot”) AND (“Capacity building” OR “training” OR “leadership development” OR “strategic planning” OR “support” )) |
| **Intervention**  **(All terms and fields)** | 13 | (#7 OR #8 OR #9 OR #10 OR #11 OR #12) |
| 1. **Outcome** | 14  Movement and Coalitions activities and achievement | (“participation” OR “Inclusion” OR “inclusivity” OR “Equitable” OR “responsive” OR “Inclusive” OR “coalition movement” OR “grassroots advocacy” OR “grassroot development” OR “community movement” ) |
|  | **15**  **Independent and Pluralistic Media** | “Diverse media” OR ((“free”) AND (“press” OR “media” OR “journalism”)) OR “civic space” OR “peaceful assembly” OR “freedom of association” OR “plurality of voices” OR “media ownership” OR “Freedom of citizens” OR “media coverage” OR “inclusive media space” OR “media corruption” OR “press freedom index” |
|  | **16**  **Use of Key Government Information** | “Corruption Perception Index” OR “budgets” OR “Global Open Data Index” OR “Rule of Law Index” OR “data governance” OR “right to information” OR “access to information” OR “use of government information” OR “government data” OR “right to information” OR “open society” OR “multi-language” OR “digitalization index” |
|  | **17**  **Policy and policymakers** | (“awareness” OR "understanding" OR "engagement") |
|  | **18**  **Capacity building outcomes** | “skills” OR “knowledge” OR “occupational capacity” |
| **Outcome**  (All terms and fields) | **19** | (#14 OR #15 OR #16 OR #17 OR #18) |
| **Study design** | **20** | “Case study” OR “impact evaluation” OR “quantitative synthesis” OR "mixed method" OR “qualitative method” OR “systematic review” OR “before vs after studies” OR “difference in difference” OR “Scoping reviews” OR “summative evaluation” OR “Modeling” OR “Process evaluation” OR “Randomized Control Trials" OR Randomised control trials” OR "regression discontinuity" OR "propensity score" OR "comparison group" OR "control group" OR "instrumental variable" OR “experimental study” OR “quasi-experiment” OR “meta-analysis” OR “field experiment” OR “descriptive” OR “regression-based study” OR “Non-experimental” |
| **Combined search** | **21**  **(#6AND #7 AND #19)** | **#6 AND #13 AND #19** |
|  |  | **#6 AND #13 AND #19 AND #20** |

**Replicable search results in SCOPUS**

**Summary results with study design**

( ( ( PUBYEAR > 1990 TITLE-ABS-KEY ( "skills" OR "knowledge" OR "occupational capacity" ) ) OR ( PUBYEAR > 1990 TITLE-ABS-KEY ( "awareness" OR "understanding" OR "engagement" ) ) OR ( PUBYEAR > 1990 TITLE-ABS-KEY ( "Corruption Perception Index" OR "budgets" OR "Global Open Data Index" OR "Rule of Law Index" OR "data governance" OR "right to information" OR "access to information" OR "use of government information" OR "government data" OR "right to information" OR "open society" OR "multi-language" OR "digitalization index" ) ) OR ( PUBYEAR > 1990 TITLE-ABS-KEY ( "Diverse media" OR ( ( "free" ) AND ( "press" OR "media" OR "journalism" ) ) OR "civic space" OR "peaceful assembly" OR "freedom of association" OR "plurality of voices" OR "media ownership" OR "Freedom of citizens" OR "media coverage" OR "inclusive media space" OR "media corruption" OR "press freedom index" ) ) OR ( PUBYEAR > 1990 TITLE-ABS-KEY ( "participation" OR "Inclusion" OR "inclusivity" OR "Equitable" OR "responsive" OR "Inclusive" OR "coalition movement" OR "grassroots advocacy" OR "grassroot development" OR "community movement" ) ) ) AND ( ( PUBYEAR > 1990 TITLE-ABS-KEY ( "movement" OR "ICT" OR "digitalization" OR "Digitalisation" OR "Capacity building" OR "Coalitions" OR "Community driven" OR "community based" OR "community" OR "Inclusive" OR "Policies" "grassroots" "campaigns" OR "organ*" OR "collective action" OR " activism" ) ) OR ( PUBYEAR > 1990 TITLE-ABS-KEY ( ( ( "CSO" OR "NGO" OR "nonprofit organi?ation" OR "Grassroot" OR "community" ) AND ( "advocacy" OR "alliance" OR "collective action" OR "lobbying" OR "campaign" ) ) ) ) OR ( PUBYEAR > 1990 TITLE-ABS-KEY ( "Inclusive governance" OR "responsive governance" OR ( ( "awareness" ) AND ( "polic" * OR "governance" ) ) ) ) OR ( PUBYEAR > 1990 TITLE-ABS-KEY ( "Gender Sensitive" OR "media spaces" OR "diversified media" OR "Gender sensitivity" OR "local radio station" ) ) OR ( PUBYEAR > 1990 TITLE-ABS-KEY ( "information access" OR "access to information" OR ( "Language" AND "translation" AND "local" ) OR "right to information" OR "liberali?ed media" OR "freedom of information" OR "open data" ) ) OR ( PUBYEAR > 1990 TITLE-ABS-KEY ( ( ( "CSO" OR "Media" OR "Policy makers" OR "grassroot" ) AND ( "Capacity building" OR "training" OR "leadership development" OR "strategic planning" OR "support" ) ) ) ) ) AND ( ( ( PUBYEAR > 1990 TITLE-ABS-KEY ( "Politicians" OR "Policy makers" OR "decision makers" OR "legislators" OR "policy advisors" OR "state actors" OR "public officers" OR "civil servant" ) ) OR ( PUBYEAR > 1990 TITLE-ABS-KEY ( ( ( "Journalists" OR "bloggers" ) AND ( "media" OR "press" OR "news" ) AND ( "radio" OR "television" OR "newspaper" ) ) ) ) OR ( PUBYEAR > 1990 TITLE-ABS-KEY ( "Non-governmental organization" OR "Civil society organization" OR "Non-profit Organizations" OR "Not-for-Profit Organizations" ) ) OR ( PUBYEAR > 1990 TITLE-ABS-KEY ( ( ( "Rural" OR "village" OR "margin*" ) AND ( "wom?n" OR "young" OR "youth" ) ) OR "Peasant farmers" OR "Small scale farmers" OR "Small holders" OR "Slum dwellers" OR "PWDs" OR "disabilit*" OR "Marginali?ed ethnic group" OR "ethnic minority" OR "sexual minority" OR "Lesbian" OR "Gay" OR "bisexual" OR "asexual" OR "transgender" OR "Two-Spirit" OR "queer" OR "intersex" OR "Fulani" OR "Fulbe" OR "fellata" OR "fula*" ) ) ) AND ( PUBYEAR > 1990 TITLE-ABS-KEY ( "Angola" OR "Burundi" OR "Central African Republic" OR "Chad" OR "Democratic Republic of Congo" OR "Republic of Congo" OR "Rwanda" OR "Comoros" OR "Eritrea" OR "Ethiopia" OR "Kenya" OR "Madagascar" OR "Mauritius" OR "Seychelles" OR "Somalia" OR "South Sudan" OR "Sudan" OR "Tanzania" OR "Uganda" OR "Botswana" OR "Eswatini" OR "Formerly Known as Swaziland" OR "Lesotho" OR "Malawi" OR "Mozambique" OR "Namibia" OR "South Africa" OR "Zambia" OR "Zimbabwe" OR "Benin" OR "Burkina Faso" OR "Cabo Verde" OR "Cape verde" OR "Cameroon" OR "Cote d'Ivoire" OR "Ivory Coast" OR "Equatorial Guinea" OR "Gabon" OR "Gambia" OR "Ghana" OR "Gold Coast" OR "Guinea" OR "Guinea Bissau" OR "Liberia" OR "Mali" OR "Mauritania" OR "Niger" OR "Nigeria" OR "Sao Tome and Principe" OR "Senegal" OR "Sierra leone" OR "Togo" OR "africa south of the sahara" OR "sub-saharan Africa" OR "subsaharan Africa" OR "africa central" OR "central Africa" OR "africa southern" OR "southern Africa" OR "africa eastern" OR "east Africa" OR "eastern Africa" OR "africa western" OR "west Africa" OR "western Africa" OR "Africa" ) ) ) ) AND ( PUBYEAR > 1990 TITLE-ABS-KEY ( "Case study" OR "impact evaluation" OR "quantitative synthesis" OR "mixed method" OR "qualitative method" OR "systematic review" OR "before vs after studies" OR "difference in difference" OR "Scoping reviews" OR "summative evaluation" OR "Modeling" OR "Process evaluation" OR "Randomized Control Trials" OR "Randomised control trials" OR "regression discontinuity" OR "propensity score" OR "comparison group" OR "control group" OR "instrumental variable" OR "experimental study" OR "quasi-experiment" OR "meta-analysis" OR "field experiment" OR "descriptive" OR "regression-based study" OR "Non-experimental" ) )

**Summary results without study**

((PUBYEAR > 1990 TITLE-ABS-KEY("skills" OR "knowledge" OR "occupational capacity")) OR (PUBYEAR > 1990 TITLE-ABS-KEY("awareness" OR "understanding" OR "engagement")) OR (PUBYEAR > 1990 TITLE-ABS-KEY("Corruption Perception Index" OR "budgets" OR "Global Open Data Index" OR "Rule of Law Index" OR "data governance" OR "right to information" OR "access to information" OR "use of government information" OR "government data" OR "right to information" OR "open society" OR "multi-language" OR "digitalization index")) OR (PUBYEAR > 1990 TITLE-ABS-KEY("Diverse media" OR (("free") AND ("press" OR "media" OR "journalism")) OR "civic space" OR "peaceful assembly" OR "freedom of association" OR "plurality of voices" OR "media ownership" OR "Freedom of citizens" OR "media coverage" OR "inclusive media space" OR "media corruption" OR "press freedom index")) OR (PUBYEAR > 1990 TITLE-ABS-KEY("participation" OR "Inclusion" OR "inclusivity" OR "Equitable" OR "responsive" OR "Inclusive" OR "coalition movement" OR "grassroots advocacy" OR "grassroot development" OR "community movement" ))) AND ((PUBYEAR > 1990 TITLE-ABS-KEY("movement" OR "ICT" OR "digitalization" OR "Digitalisation" OR "Capacity building" OR "Coalitions" OR "Community driven" OR "community based" OR "community" OR "Inclusive" OR "Policies" "grassroots" "campaigns" OR "organ*" OR "collective action" OR " activism")) OR (PUBYEAR > 1990 TITLE-ABS-KEY((("CSO" OR "NGO" OR "nonprofit organi?ation" OR "Grassroot" OR "community") AND ("advocacy" OR "alliance" OR "collective action" OR "lobbying" OR "campaign")))) OR (PUBYEAR > 1990 TITLE-ABS-KEY("Inclusive governance" OR "responsive governance" OR (("awareness") AND ("polic"* OR "governance")))) OR (PUBYEAR > 1990 TITLE-ABS-KEY("Gender Sensitive" OR "media spaces" OR "diversified media" OR "Gender sensitivity" OR "local radio station")) OR (PUBYEAR > 1990 TITLE-ABS-KEY("information access" OR "access to information" OR ("Language" AND "translation" AND "local") OR "right to information" OR "liberali?ed media" OR "freedom of information" OR "open data")) OR (PUBYEAR > 1990 TITLE-ABS-KEY((("CSO" OR "Media" OR "Policy makers" OR "grassroot") AND ("Capacity building" OR "training" OR "leadership development" OR "strategic planning" OR "support" ))))) AND (((PUBYEAR > 1990 TITLE-ABS-KEY("Politicians" OR "Policy makers" OR "decision makers" OR "legislators" OR "policy advisors" OR "state actors" OR "public officers" OR "civil servant" )) OR (PUBYEAR > 1990 TITLE-ABS-KEY((("Journalists" OR "bloggers") AND ("media" OR "press" OR "news") AND ("radio" OR "television" OR "newspaper")))) OR (PUBYEAR > 1990 TITLE-ABS-KEY("Non-governmental organization" OR "Civil society organization" OR "Non-profit Organizations" OR "Not-for-Profit Organizations")) OR (PUBYEAR > 1990 TITLE-ABS-KEY((("Rural" OR "village" OR "margin*") AND ("wom?n" OR "young" OR "youth")) OR "Peasant farmers" OR "Small scale farmers" OR "Small holders" OR "Slum dwellers" OR "PWDs" OR "disabilit*" OR "Marginali?ed ethnic group" OR "ethnic minority" OR "sexual minority" OR "Lesbian" OR "Gay" OR "bisexual" OR "asexual" OR "transgender" OR "Two-Spirit" OR "queer" OR "intersex" OR "Fulani" OR "Fulbe" OR "fellata" OR "fula*"))) AND (PUBYEAR > 1990 TITLE-ABS-KEY("Angola" OR "Burundi" OR "Central African Republic" OR "Chad" OR "Democratic Republic of Congo" OR "Republic of Congo" OR "Rwanda" OR "Comoros" OR "Eritrea" OR "Ethiopia" OR "Kenya" OR "Madagascar" OR "Mauritius" OR "Seychelles" OR "Somalia" OR "South Sudan" OR "Sudan" OR "Tanzania" OR "Uganda" OR "Botswana" OR "Eswatini" OR "Formerly Known as Swaziland" OR "Lesotho" OR "Malawi" OR "Mozambique" OR "Namibia" OR "South Africa" OR "Zambia" OR "Zimbabwe" OR "Benin" OR "Burkina Faso" OR "Cabo Verde" OR "Cape verde" OR "Cameroon" OR "Cote d'Ivoire" OR "Ivory Coast" OR "Equatorial Guinea" OR "Gabon" OR "Gambia" OR "Ghana" OR "Gold Coast" OR "Guinea" OR "Guinea Bissau" OR "Liberia" OR "Mali" OR "Mauritania" OR "Niger" OR "Nigeria" OR "Sao Tome and Principe" OR "Senegal" OR "Sierra leone" OR "Togo" OR "africa south of the sahara" OR "sub-saharan Africa" OR "subsaharan Africa" OR "africa central" OR "central Africa" OR "africa southern" OR "southern Africa" OR "africa eastern" OR "east Africa" OR "eastern Africa" OR "africa western" OR "west Africa" OR "western Africa" OR "Africa" )))

| **Appendix 2: Coding Form**  **Inclusive Governance Evidence and Gap Map Coding Form** | | |
| --- | --- | --- |
|  | **Parent code** | **Child code** |
| **Language** | English |  |
|  | French |  |
| **Region** | Central Africa |  |
|  | Eastern Africa |  |
|  | Western Africa |  |
|  | Southern Africa |  |
| **Country** | Angola |  |
|  | Burundi |  |
|  | Central African Republic |  |
|  | Chad |  |
|  | Democratic Republic of Congo |  |
|  | Republic of Congo |  |
|  | Rwanda |  |
|  | Comoros |  |
|  | Eritrea |  |
|  | Ethiopia |  |
|  | Kenya |  |
|  | Madagascar |  |
|  | Mauritius |  |
|  | Seychelles |  |
|  | Somalia |  |
|  | South Sudan |  |
|  | Sudan |  |
|  | Tanzania |  |
|  | Uganda |  |
|  | Botswana |  |
|  | Eswatini (Formerly Known as Swaziland) |  |
|  | Lesotho |  |
|  | Malawi |  |
|  | Mozambique |  |
|  | Namibia |  |
|  | South Africa |  |
|  | Zambia |  |
|  | Zimbabwe |  |
|  | Benin |  |
|  | Burkina Faso |  |
|  | Cabo Verde |  |
|  | Cameroon |  |
|  | Cote d'Ivoire |  |
|  | Equatorial Guinea |  |
|  | Gabon |  |
|  | Gambia, The |  |
|  | Ghana |  |
|  | Guinea |  |
|  | Guinea-Bissau |  |
|  | Liberia |  |
|  | Mali |  |
|  | Mauritania |  |
|  | Niger |  |
|  | Nigeria |  |
|  | Sao Tome and Principe |  |
|  | Senegal |  |
|  | Sierra Leone |  |
|  | Togo |  |
| **Study status** | Completed |  |
|  | Ongoing |  |
| **Study population** | Underserved populations | Peasant/smallholder farmers |
|  |  | Women (Women from rural areas, ethnic minority communities, and informal settlements in urban areas including market women); |
|  |  | Youth (Young boys and girls from rural areas, urban informal settlements, and young girls from ethnic minority communities) |
|  |  | Persons Living with Disabilities (PWDs) |
|  |  | Sexual minorities |
|  |  | Fulbe Community |
|  | CSO’s |  |
|  | Journalists |  |
|  | Politicians |  |
| **Intervention** | Grassroots movement | Developing and strengthening grassroots movements and coalitions |
|  |  | Mobilizing resources for movements and coalitions |
|  |  | Collective actions and campaigns |
|  | CSOs’ advocacy | Developing local philanthropic networks and initiatives |
|  |  | Alliance building, advocacy and lobbying |
|  |  | Active participation of women and youth in coalitions and movements |
|  | **Public policy and government agencies** | Inclusive and responsive governance frameworks |
|  |  | Creating awareness of public policies and governance mechanisms |
|  | **Media** | Liberalization of media spaces |
|  |  | Diversified media programs |
|  |  | Elevation of voices of underserved populations |
|  |  | Gender sensitivity culture in media |
|  | Access to information | Accessibility of local and national information by underserved populations |
|  |  | Official information and documents available in local languages |
|  |  | Right to information policy |
|  | **Capacity building initiatives** | Capacity building for grassroots and CSOs |
|  |  | Capacity building initiatives for national, public and local-level agencies |
|  |  | Capacity-building and direct support for media personnel and journalists |
| **Outcome** | **Movement and Coalitions, & CSO’s activities and achievement** | Funding to movements and coalitions |
|  |  | Participation of local  communities in the activities and campaigns of movements and coalitions |
|  |  | Grassroots and CSOs influence on public policies |
|  |  | Civic awareness |
|  |  | Partnerships |
|  | **Civic participation** | Civic participation in coalitions and movements |
|  |  | Civic participation in governance |
|  |  | Social, cultural and political change |
|  | **Capacity** | Capacity of grassroots movements, CSOs, media and policy makers |
|  | **Independent and Pluralistic Media** | Enhanced Civic Space |
|  |  | Media coverage of undeserved groups |
|  | **Use of Key Government Information** | Global Right to Information Rating |
|  |  | Rule of Law Index |
|  |  | Public participation in budgets |
|  |  | Corruption Perception Index |
|  |  | Global Open Data Index |
|  | **Policy and policymakers** | Knowledge, attitude, or behavior of policymakers, the public,  or other key actors |
| **Study design** | **Experimental** | Experimental study |
|  | **Non-experimental** | Non-experimental (quasi-experiment, descriptive and regression based study) |
|  | **Qualitative** | Case study |
|  |  | Other qualitative |
|  | **Modeling studies** | Modeling studies |
|  | **Systematic review** | Systematic reviews |
|  | **Scoping reviews** | Scoping reviews |
| **Evaluation types** | Impact evaluation |  |
|  | Process evaluation |  |
|  | Formative evaluation |  |
|  | Summative evaluation |  |
| **Publication type** | Peer review article |  |
|  | Pre-print peer review article |  |
|  | Report |  |
|  | Conference paper |  |
|  | Working paper |  |
|  | Discussion paper |  |
|  | Dissertation |  |
|  | Protocol |  |
| **Year of publication** | 1990 |  |
|  | 1991 |  |
|  | 1992 |  |
|  | 1993 |  |
|  | 1994 |  |
|  | 1995 |  |
|  | 1996 |  |
|  | 1997 |  |
|  | 1998 |  |
|  | 1999 |  |
|  | 2000 |  |
|  | 2001 |  |
|  | 2002 |  |
|  | 2003 |  |
|  | 2004 |  |
|  | 2005 |  |
|  | 2006 |  |
|  | 2007 |  |
|  | 2008 |  |
|  | 2009 |  |
|  | 2010 |  |
|  | 2011 |  |
|  | 2012 |  |
|  | 2013 |  |
|  | 2014 |  |
|  | 2015 |  |
|  | 2016 |  |
|  | 2017 |  |
|  | 2018 |  |
|  | 2019 |  |
|  | 2020 |  |
|  | 2021 |  |
|  | 2022 |  |
|  | 2023 |  |
